# Data of continuous harvest of stem cells via partial detachment from thermoresponsive nanobrush surfaces

**DOI:** 10.1016/j.dib.2015.12.056

**Published:** 2016-01-13

**Authors:** Chin-Chen Yeh, Saradaprasan Muduli, I-Chia Peng, Yi-Tung Lu, Qing-Dong Ling, Abdullah A. Alarfaj, Murugan A. Munusamy, S. Suresh Kumar, Kadarkarai Murugan, Da-Chung Chen, Hsin-chung Lee, Yung Chang, Akon Higuchi

**Affiliations:** a Department of Chemical Engineering, R&D Center for Membrane Technology, Chung Yuan Christian University, 200, Chung-Bei Rd., Chungli, Taoyuan 320, Taiwan; b Department of Chemical and Materials Engineering, National Central University, No. 300, Jhongda Rd., Jhongli, Taoyuan 32001, Taiwan; c Cathay Medical Research Institute, Cathay General Hospital, No. 32, Ln 160, Jian-Cheng Road, Hsi-Chi City, Taipei 221, Taiwan; d Institute of Systems Biology and Bioinformatics, National Central University, No. 300, Jhongda Rd., Jhongli, Taoyuan 32001, Taiwan; e Department of Botany and Microbiology, King Saud University, Riyadh 11451, Saudi Arabia; f Department of Medical Microbiology and Parasitology, Universiti Putra Malaysia, Serdang 43400, Selangor, Malaysia; g Division of Entomology, Department of Zoology, School of Life Sciences, Bharathiar University, Coimbatore, Tamil Nadu 641046, India; h Department of Obstetrics and Gynecology, Taiwan Landseed Hospital, 77, Kuangtai Road, Pingjen City, Taoyuan 32405, Taiwan; i Department of Surgery, Cathay General Hospital, No.280, Sec. 4, Ren’ai Rd., Da’an Dist., Taipei 10693, Taiwan; j Graduate Institute of Translational and Interdisciplinary Medicine, College of Health Science and Technology, National Central University, No. 300, Jhongda Rd., Jhongli, Taoyuan 32001, Taiwan

## Abstract

This data article contains two figures and one table supporting the research article entitled: “Continuous harvest of stem cells via partial detachment from thermoresponsive nanobrush surface” [1]. The table shows coating conditions of three copolymers, poly(styrene-co-acrylic acid) grafted with oligovitronectin, poly(styrene-co-N-isopropylacrylamide) and poly(styrene-co-polyethylene glycol methacrylate) to prepare thermoresponsive surface. XPS spectra show the nitrogen peak of the polystyrene surface coated with poly(styrene-co-acrylic acid) grafted with oligovitronectin. The surface coating density analyzed from sorption of poly(styrene-co-acrylic acid) grafted with oligovitronectin by UV–vis spectroscopy is also presented.

## Specifications table

**Table:** 

Subject area	Chemistry
More specific subject area	Biomaterials
Type of data	Table, figure
How data was acquired	XPS, UV–vis spectroscopy
Data format	Analyzed
Experimental factors	Poly(styrene-co-acrylic acid) grafted with oligovitronectin was coated on tissue culture polystyrene dishes
Experimental features	See experimental details for each figure
Data source location	Taiwan
Data accessibility	Within this article

## Value of the data

• The data show coating conditions of three copolymers, poly(styrene-co-acrylic acid) grafted with oligovitronectin, poly(styrene-co-N-isopropylacrylamide) and poly(styrene-co-polyethylene glycol methacrylate) on polystyrene tissue culture plates for the preparation of thermoresponsive surface.
• The data show which concentration of the coating polymer is necessary to cover the surface.
• The surface coating density can be measured by spectroscopy on the surface coated with poly(styrene-co-acrylic acid) grafted with oligovitronectin
• The data show the evaluation of oligovitronectin measured by XPS spectra of the surface coated with poly(styrene-co-acrylic acid) grafted with oligovitronectin
• The existence of oligovitronectin on the surface coated with poly(styrene-co-acrylic acid) can be verified by XPS measurement.

## Data

Table 1 shows coating conditions of three copolymers, (a) poly(styrene-co-acrylic acid) grafted with oligovitronectin (P[St-AA]-oligoVN), (b) poly(styrene-co-N-isopropylacrylamide) (P[St-PNIPAAm]) and (c) poly(styrene-co-polyethylene glycol methacrylate) (P[St-PEGMA]) to prepare thermoresponsive surface.

**Fig. 1:**
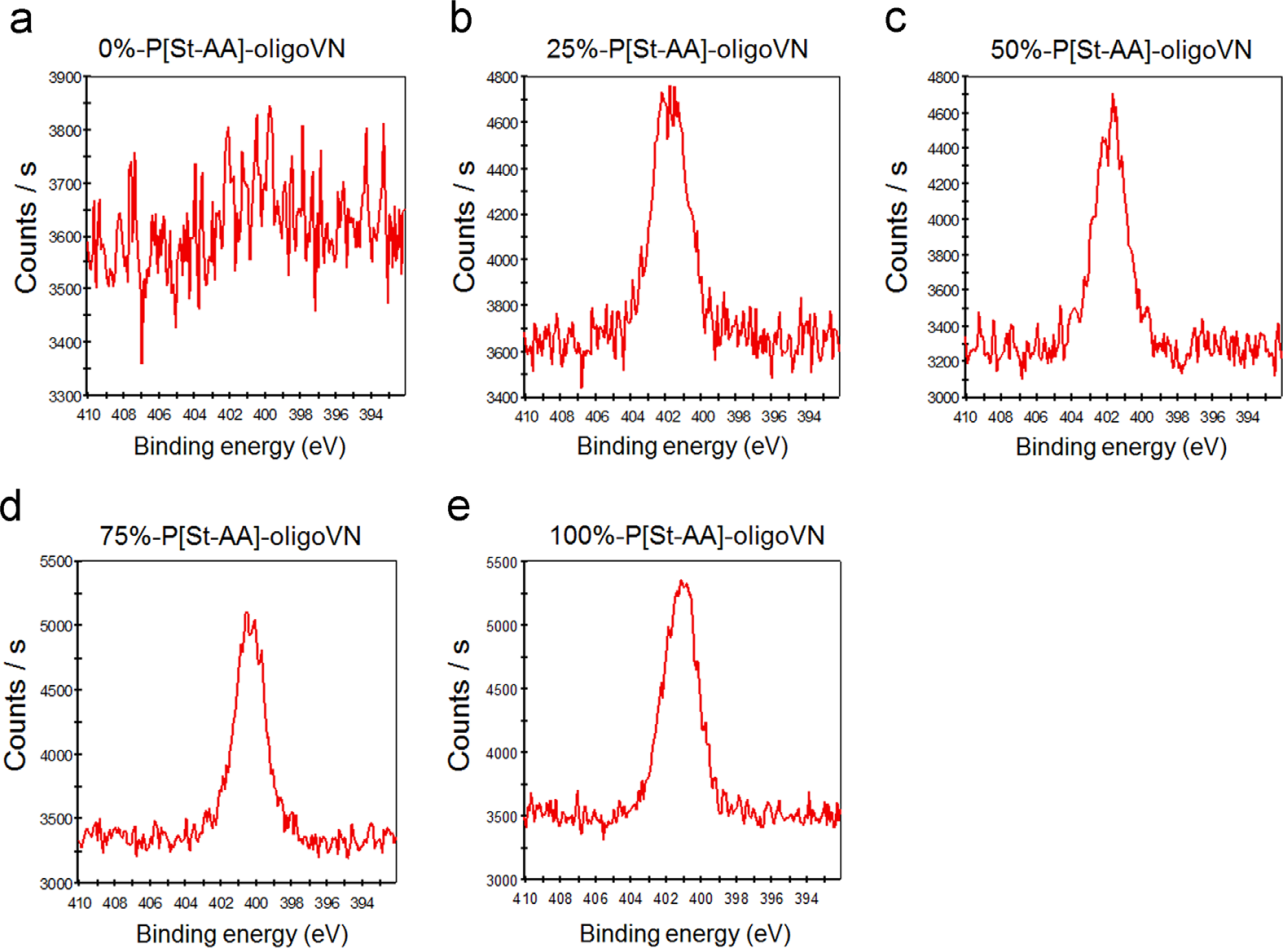
High-resolution XPS spectra of the N1s peaks obtained on the surface of 0% (a), 25% (b), 50% (c), 75% (d), and 100% (e) of surface coverage of P[St-AA]-oligoVN. Nitrogen atoms originated from oligoVN on the surface [(b)–(e)], whereas no nitrogen atoms were observed on the non-coated TCPS surface (a).

Fig. 1 shows high-resolution X-ray photoelectron spectroscopy (XPS) spectra of the N1s peaks obtained on the surface of 0% (a), 25% (b), 50% (c), 75% (d), and 100% (e) of surface coverage of P[St-AA]-oligoVN where the surface coverage % of P[St-AA]-oligoVN is defined as % adsorption of [St-AA]-oligoVN on the surface from the saturated adsorption amount (500 μg/cm^2^ for 100%, 375 μg/cm^2^ for 75%, 250 μg/cm^2^ for 50%, and 125 μg/cm^2^ for 25%). Nitrogen atoms originated from oligoVN on the surface [(b)-(e)], whereas no nitrogen atoms were observed on the non-coated tissue culture polystyrene (TCPS) surface (a)

**Fig. 2:**
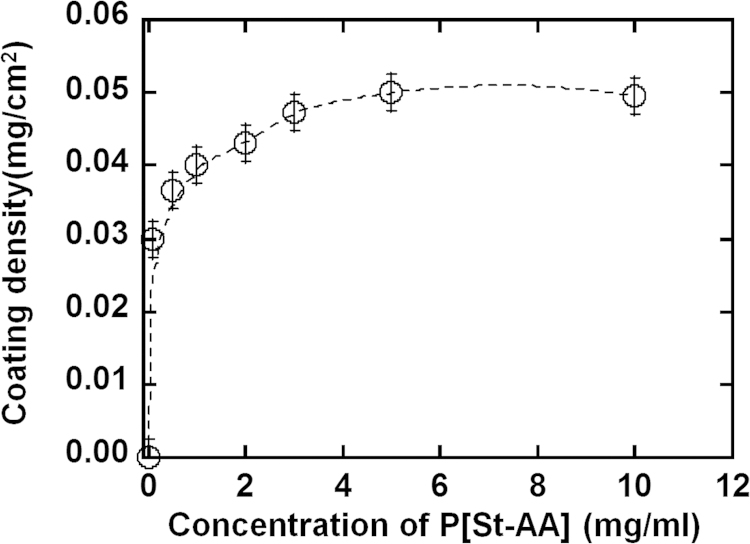
Dependence of coating density of P[St-AA]-oligoVN on the concentration of coating solution. Coating density was measured by the decrease of optical density of coating solution of P[St-AA]-oligoVN after immersion of TCPS plates into the solution.

Fig. 2 shows dependence of surface coating density of P[St-AA]-oligoVN on the concentration of coating solution. Coating density was measured by the decrease of optical density of coating solution of P[St-AA]-oligoVN after immersion of TCPS plates into the coating solution.

## 1. Experimental design, materials and methods

We designed three types of coating copolymers: (a) a stem cell binding site, (b) a thermoresponsive site, and (c) a hydrophilic site. Hydrophobic polystyrene (PSt) was selected as the anchoring site of these three copolymers on the surface of TCPS. For this purpose, we synthesized three copolymers (a) P[St-AA]-oligoVN having the stem cell binding site of oligoVN (amino acid sequence of KGGPQVTRGDVFTMP) [2], (b) P[St-NIPAAm] having thermoresponsive polyNIPAAm [3] and (c) P[St-PEGMA] having hydrophilc PEGMA to prepare thermoresponsive surface.

### 1.1. Synthesis of copolymers

P[St-AA], P[St-NIPAAm], and P[St-PEGMA] were prepared by a reversible addition-fragmentation chain transfer (RAFT) polymerization. The synthesis method of these copolymers was described in Ref. [1] in detail.

### 1.2. Preparation process of thermoresponsive nanobrush surface

0–3 mg/mL of P[St-AA] in ethanol was added in TCPS dishes (4 cm^2^ of surface area, 12 well dishes) for coating of P[St-AA] on the surface for 2 h at 25 °C and subsequently removed from the dishes. TCPS dishes coated with P[St-AA] were activated via immersion in an aqueous solution containing 10 mg/ml N-(3-dimethylaminopropyl)-N’-ethylcarbodiimide hydrochloride (EDC) and 10 mg/ml N-hydroxysuccinimide (NHS) for 1 h at 37 °C after washing the dishes with phosphate buffered saline (PBS, pH 7.2) three times [1], [4], [5]. Subsequently, the dishes were washed with PBS and immersed in a PBS solution containing 1000 μg/mL of oligoVN for 24 h at 4 °C to prepare P[St-AA]-oligoVN dishes. The dishes were washed with PBS three times [1].

### 1.3. Characterization of dishes by XPS

The chemical composition of the dishes on the TCPS surface with P[St-AA]-oligoVN was analyzed using X-ray photoelectron spectroscopy (XPS, K-Alpha spectrometer, Thermal Scientific, Inc., Amarillo, TX, USA, equipped with a monochromatic Al-K X-ray source [1486.6 eV photons]). The energy of the emitted electrons was measured using a hemispherical energy analyzer at pass energies ranging from 50 to 150 eV. Data were collected at a photoelectron takeoff angle of 45° with respect to the sample surface. The binding energy (BE) scale was referenced by setting the peak maximum in the C1s spectrum to 284.6 eV. The obtained high-resolution C1s spectra were fitted using Shirley background subtraction and a series of Gaussian peaks [1], [5].

## Figures and Tables

**Table 1:** Coating conditions of thermoresponsive surface used in this study.

Coating conditions	Coating concentration (mg/ml)			Figure no. used in Ref. [1]
P[St-AA]	P[St-PNIPAAm]	P[St-PEG]
25% surface coverage of P[St-AA]-oligoVN	0.750	0	0	Fig. 4 A
25% surface coverage of P[St-AA]-oligoVN with 1:0=P[St-NIPAAm]: P[St-PEG]	0.750	3.000	0	Fig. 2A, 2D, 4A–D
25% surface coverage of P[St-AA]-oligoVN with 9:1=P[St-NIPAAm]: P[St-PEG]	0.750	2.700	0.300	Fig. 4B, 4 C, and 4D
25% surface coverage of P[St-AA]-oligoVN with 4:1=P[St-NIPAAm]: P[St-PEG]	0.750	2.400	0.600	Figs. 2A, C–E, 3, 4A–D
25% surface coverage of P[St-AA]-oligoVN with 7:3=P[St-NIPAAm]: P[St-PEG]	0.750	2.100	0.900	Fig. 2A and D
25% surface coverage of P[St-AA]-oligoVN with 0:1=P[St-NIPAAm]: P[St-PEG]	0.750	0	3.000	Fig. 2A, 4B–D
50% surface coverage of P[St-AA]-oligoVN with 1:0=P[St-NIPAAm]: P[St-PEG]	1.500	3.000	0	Fig. 4B–D
50% surface coverage of P[St-AA]-oligoVN with 9:1=P[St-NIPAAm]: P[St-PEG]	1.500	2.700	0.300	Fig. 4B–D
50% surface coverage of P[St-AA]-oligoVN with 4:1=P[St-NIPAAm]: P[St-PEG]	1.500	2.400	0.600	Fig. 4B–D
50% surface coverage of P[St-AA]-oligoVN with 0:1=P[St-NIPAAm]: P[St-PEG]	1.500	0	3.000	Fig. 4B–D
75% surface coverage of P[St-AA]-oligoVN with 1:0=P[St-NIPAAm]: P[St-PEG]	2.250	3.000	0	Fig. 4B–D
75% surface coverage of P[St-AA]-oligoVN with 9:1=P[St-NIPAAm]: P[St-PEG]	2.250	2.700	0.300	Fig. 4B–D
75% surface coverage of P[St-AA]-oligoVN with 4:1=P[St-NIPAAm]: P[St-PEG]	2.250	2.400	0.600	Fig. 4B–D
75% surface coverage of P[St-AA]-oligoVN with 0:1=P[St-NIPAAm]: P[St-PEG]	2.250	0	3.000	Fig. 4B–D
100% surface coverage of P[St-AA]-oligoVN with 1:0=P[St-NIPAAm]: P[St-PEG]	3.000	3.000	0	Fig. 4B–D
100% surface coverage of P[St-AA]-oligoVN with 9:1=P[St-NIPAAm]: P[St-PEG]	3.000	2.700	0.300	Fig. 4–D
100% surface coverage of P[St-AA]-oligoVN with 4:1=P[St-NIPAAm]: P[St-PEG]	3.000	2.400	0.600	Fig. 4–D, 5B–D, and 6
100% surface coverage of P[St-AA]-oligoVN with 0:1=P[St-NIPAAm]: P[St-PEG]	3.000	0	3.000	Fig. 4–D
